# Effect of In Vitro Culture of Long Shoot Tip on Variant Structure and Titer of Grapevine Viruses

**DOI:** 10.3390/plants11151907

**Published:** 2022-07-23

**Authors:** Guojun Hu, Yafeng Dong, Zunping Zhang, Xudong Fan, Fang Ren

**Affiliations:** Research Institute of Pomology, Chinese Academy of Agricultural Sciences, Xingcheng 125100, China; huguojun@caas.cn (G.H.); zhangzunping@caas.cn (Z.Z.); fanxudong@caas.cn (X.F.); renfang@caas.cn (F.R.)

**Keywords:** grapevine, long shoot tip culture, grapevine viruses, virus variant structure, viral titer

## Abstract

Shoot tip culture is a very effective approach for studying plant viruses. In this study, we evaluated the numbers, diversity, and titer of grapevine viruses in in vitro grapevine plants after long shoot tip culture. Six virus-infected grapevine cultivars (Cabernet Franc, Cabernet Gernischt, Cabernet Sauvignon, Wink, Victoria, and Merlot) collected from six regions of China were used as the research materials. Approximately 1.5 cm long shoot tips were used for meristem culture. The average survival rate of the six grapevine cultivars was 45.7%. Merlot collected from Beijing showed the highest survival rate (80.0%). Regeneration was not achieved in Cabernet Gernischt collected from Liaoning province and Cabernet Sauvignon from Tianjin due to bacterial and fungal contamination. Virus detection conducted in the surviving regenerated plants showed that the virus infection status, including the viral numbers and the species present in plants grown in vitro, was the same as that in corresponding in vivo plants. Moreover, the analysis of sequence diversity and the mutation frequency in grapevine viruses in vitro indicated that the structure of grapevine viruses was stable in long shoot tip culture after four sub-culture passages. Further, the relative viral titer of in vitro grapevine plants was much higher than that of in vivo plants. These results aid in the investigation of viruses in woody plants.

## 1. Introduction

Plant tissue culture is indispensable for the rapid multiplication of rare plants, plant genetic transformation, and production of plant-derived metabolites of important commercial value [1]. Modern plant tissue culture techniques increase the variety of cultures and provide a new platform for in vitro studies of plant cells. Compared to plants grown in the field, in vitro plants can acquire a better environmental containment. Generally, the production times are shorter for in vitro plants, the system is easily regulated, and proteins secreted from the cells, downstream processing and product purification are simpler and cheaper [2].

Tissue culture, such as meristem culture of shoot tips, is a very effective approach to obtain virus-free plants from a wide range of hosts. It can maximize the propagation of the sterile stocks suitable for certification schemes [3]. Some research has shown that shoot tip culture alone remained insufficient for the elimination of some plant viruses [4,5]. Therefore, shoot tip culture is usually combined with thermotherapy, chemotherapy, or cryotherapy to eliminate viruses [6,7,8].

Plant tissue culture also contributes substantially to exploring viral replication and virus–host interaction, and provides the basic methodology to generate virus resistant plants through genetic engineering [9,10,11]. Many woody plant viruses cannot be mechanically transmitted to herbaceous hosts, which is hampered by gene expression strategies and replication. Plant tissue culture techniques can be helpful to overcome these limitations [12]. The successful viral elimination of meristem tip culture depends on the size of the explant, and a smaller tip (<1 mm) could lead to a higher death rate [13,14].

In fact, large tissues can also be cultured, if only to obtain in vitro plants, and it is much easier to obtain a big tip than a small one. Studies have found that smaller shoot tip culture can remove viruses from infected plants [15,16,17], but the effect of longer shoot tip culture on viruses is unclear. Grapevine is a fruit crop with major relevance worldwide and is also among the most well-studied deciduous woody perennials. Viral diseases are major constraints to grape production worldwide. Nearly 90 virus species infect grapevine, and most of them cannot be inoculated to herbs by mechanical transmission from sap, which has seriously influenced the pathogenicity research of grapevine viruses [18,19,20,21]. Moreover, small grapevine shoot tips are commonly used as material for elimination treatment to obtain virus-free grapevine plants [22,23,24]. Meanwhile, as deciduous fruit trees, in vitro virus-infected plants are of great significance in the study of grapevine viruses. In vitro culture of long shoot tip is an effective way to achieve the above mentioned goal. However, the effect of long shoot tip culture on viruses is unclear. In this study, the numbers, diversity, and titer of viruses in in vitro grapevine plants were assessed after long shoot tip culture.

## 2. Results

### 2.1. Survival of Shoot Tips

**Figure 1:**
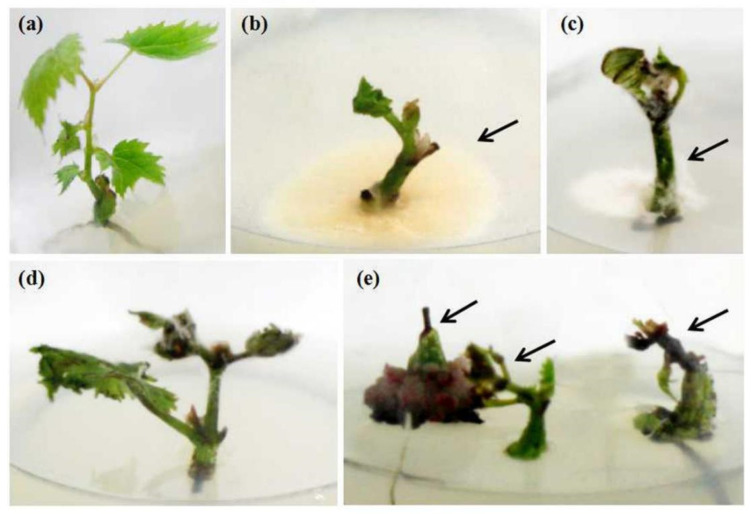
Factors affecting the survival of long shoot tip culture. (**a**) Survival regenerated grapevine plants; (**b**) Contamination of bacteria; (**c**,**d**) Contamination of fungi; (**e**) Callus of shoot tip base and browning of shoot tip top.

The survival rate was calculated after the shoot tip regenerated into a whole plant (Figure 1a). The average survival rate of six grapevine cultivars from six regions was 45.7%. Merlot collected from Beijing showed the highest survival rate of 80.0%. Regeneration was not observed in Cabernet Gernischt from Liaoning province and Cabernet Sauvignon from Tianjin. Other grapevine cultivars showed 50.0–70.0% survival rates (Table 1). There were two reasons related to shoot tip death: bacterial and fungal contamination owing to inadequate sterilization time (Figure 1b–d) and the highly differentiated callus on the base of the shoot tip, which caused the gradual browning, blackening, and wilting of meristem on the top (Figure 1e).

### 2.2. Virus Detection of In Vitro Grapevine Plantlets

**Figure 2:**
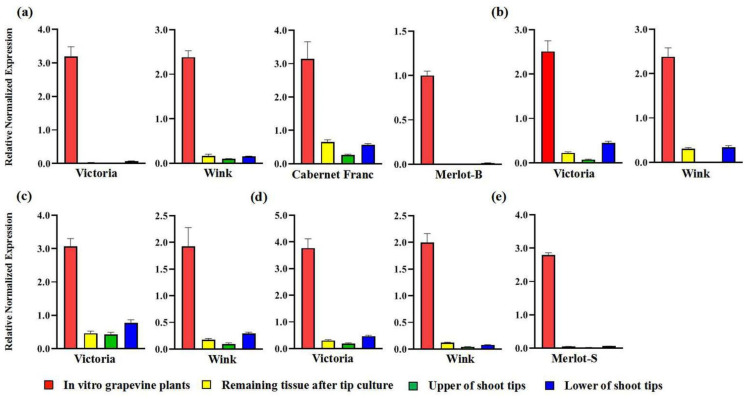
The relative concentrations of (**a**) GRSPaV, (**b**) GRLaV-3, (**c**) GFkV, (**d**) GRLaV-2, and (**e**) GRLaV-1 in different materials of different grapevine cultivars.

**Figure 3:**
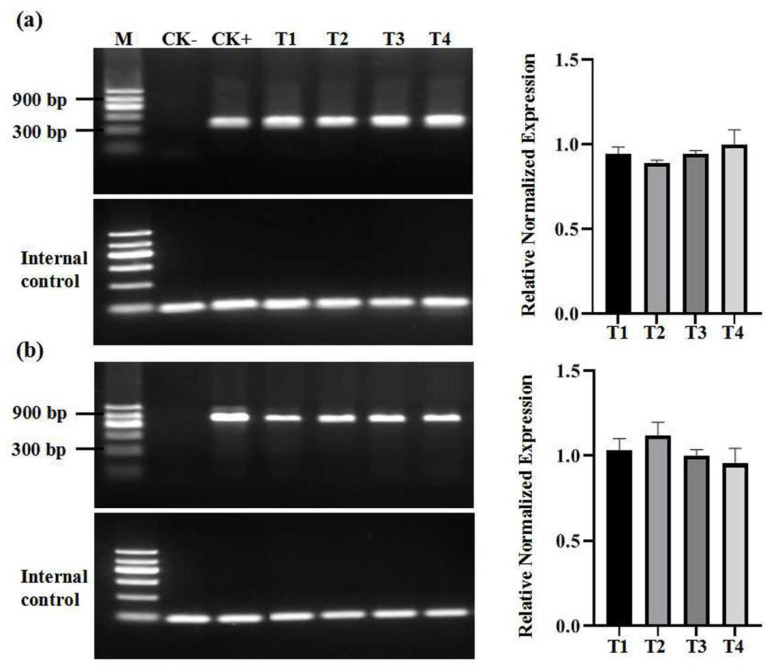
Assays of (**a**) GRLaV-2 and (**b**) GRSPaV in in vitro sub-culture of Cabernet Franc. M: maker II (Tiangen, China); CK-: negative control; CK+: positive control; T1-T4: passage number of in vitro sub-culture.

Both Wink and Victoria from Guangxi province were infected by grapevine rupestris stem pitting-associated virus (GRSPaV), grapevine leafroll-associated virus 2 (GLRaV-2), grapevine leafroll-associated virus 3 (GLRaV-3), and grapevine fleck virus. Cabernet Franc from Ningxia and Merlot from Sichuan province were infected by two viruses (Table 1). Only one virus (GRSPaV) was detected in Merlot of Beijing. These viruses (GRSPaV, GLRaV-1, GLRaV-2, GLRaV-3, GFkV, and GVA) have been found to be present and common grapevine viruses in China [25,26]. The virus infection status, including the viral numbers and the species of in vitro plants, was the same as that of the corresponding in vivo plants (Table 1). For long shoot tips, the virus titer in the upper part of shoot tips was relatively lower than that in the lower part of shoot tips, while the relative viral titer of plants regenerated from the upper part of shoot tips was substantially higher than that of the remaining tissue after tip culture (Figure 2). Moreover, we also analyzed the viral titer in four sub-cultures of Cabernet Franc from Ningxia and observed that the titer of grapevine viruses was relatively stable and that no significant change was found during sub-culture (Figure 3).

### 2.3. Diversity of In Vitro Grapevine Plantlets

The diversity of GRLaV-2, GRLaV-3, and GRSPaV in grapevine cultivars was analyzed before and after tissue culture. The products of the viruses amplified by regular PCR were used to clone and sequence. The nucleotide identities of GRLaV-2 sequence variants isolated from Wink, Victoria, and Cabernet Franc shoot tips were 99.7–100.0%, 99.5–100.0%, and 98.9–100.0%, respectively. After tissue culture, the identities of sequence variants from the three GRLaV-2 isolates were 99.4–100.0%, 99.2–100.0%, and 99.5–100.0%, respectively. The two groups of the three cultivars showed 99.4–100.0%, 99.2–100.0%, and 98.9–100.0% sequence identities and 99.97%, 99.90%, and 99.73% consistencies, respectively. The GRLaV-3 sequence variants isolated from Wink and Victoria, and the GRSPaV sequence variants isolated from Wink and Merlot (Beijing, Merlot-B) also showed similar results (Table 2). Based on the taxonomic system related to the genera Closterovirus, Ampelovirus, and Foveavirus, these results indicated that the sequence diversity of grapevine viruses hardly changed during shoot tip culture.

To further demonstrate the stability of grapevine virus population composition during shoot tip culture, we also computed the mutation frequency. Table 2 lists the mutation frequency/nt of GRLaV-2, GRLaV-3, and GRSPaV in different cultivars before and after shoot tip culture. The nucleotide mutation frequencies of the GRLaV-2 sequence isolated from Cabernet Franc, the GRLaV-3 sequence isolated from Wink, and the GRSPaV sequence isolated from Merlot-B were the same for in vivo and in vitro plants. The nucleotide mutation frequency of the GRLaV-2 sequence isolated from in vitro Victoria was lower than that detected from in vivo plants, which demonstrated that the diversity of the population structure of GRLaV-2 scarcely changed after shoot tip culture. The nucleotide mutation frequencies of the GRLaV-2 and GRSPaV sequences isolated from in vitro Wink were 1.8 × 10^−4^ and 0.8 × 10^−4^ more than those from in vivo plants, respectively, but it was found that the two mutations were not produced during virus replication after they were revised by error rate (Table 2). These results also indicated that the structure of grapevine viruses was stable after long shoot tip culture.

## 3. Discussion

In the current study, the infection status and genetic diversity of viruses were the same in in vitro and in vivo grapevine plants after long shoot tip culture. Meanwhile, the viral titer markedly increased after shoot tip culture.

Most research has demonstrated that the size of shoot tips is directly related to the survival rate of cultures, and that smaller sizes could led to a higher death rate [13,14]. Han et al. [22] demonstrated that the survival rate of small shoot tips (<0.5 mm) was less than 65%. Moreover, previously we reported that the survival rate was related to the cultivars of grapevine [23,24]. Here, we observed that the extension of shoot tips (≈1.5 cm) did not significantly improve the culture survival rate. Abundant parasitic or infective fungi and bacteria existed on the surface and inside the new and tender long shoot tips. The timing of ethanol and mercury bichloride treatments should be optimized in order to select the suitable treatment for long shoot tips specifically.

Plant tissue culture has a wide range of uses in modern biotechnology. The regeneration abilities of different parts of explants are different [10,27,28]. We found that a large number of calli formed at the base of long shoot tips, but these calli were unable to regenerate. Moreover, the production of calli blocked the supply of nutrition and water, resulting in the death of newly differentiated leaves or apical tips. The callus development was closely correlated with the activity of accumulated auxins at the basal cut ends, which stimulates cell proliferation [29,30,31]. Therefore, regulating the concentration of the hormone could be helpful to increase the survival rate of the long shoot tips of grapevine.

RNA viruses have high mutation rates owing to the mismatch recognition of RNA-dependent RNA polymerase, which has been found to lead to increases in genetic diversity and evolutionary change [32]. Genetic variation is generated at the first replication cycle of a viral clone and unique viral lineages, and separate evolutionary trajectories might be created during the consecutive infection cycles [33]. It has been reported that the high levels of genetic diversity in plant viruses are linked to their ability to adapt to changing environments [34]. Our results demonstrated that even in the changed infection environment in shoot tip culture, the genetic diversity of grapevine viruses was unchanged. In addition, previous research proved that viral diversity was stable during sub-culture [35]. The accumulation of differences may be a slow process, and individual isolates of many plant viruses have shown low levels of variation [36]. Long-term monitoring can confirm this fact.

Different tissue culture techniques are of great significance for research on plant viruses. The viral replication and virus–host interaction of tobacco mosaic virus, potato spindle tuber viroid, and turnip yellow mosaic virus have been explored through callus culture, cell culture, and cell suspension culture of herbaceous host plants [9,37,38]. Svensson et al. [12] studied the genome replication and gene expression of European mountain ash ringspot-associated virus (EMARaV) in Sorbus aucuparia and found that the EMARaV infection of calli and cell suspension cultures was detectable even 18 months after callus induction. In this study, we demonstrated that long shoot tip culture could increase the titers of grapevine viruses. Viruses in woody plants often have low titers, which seriously affects research into their detection, replication and interaction with hosts. Our result is helpful for the above mentioned research. AlKhazindar et al. [28] found that virus elimination using small shoot tips (<0.5 mm) was related to the action of growth regulators, cell injury during excision, poor development of vascular tissue, and the descending virus concentration from the base of the plant towards the meristems. Bradamante [39] suggested that apical meristems possess effective antiviral barriers that prevent many pathogenic viruses from entering and/or establishing infection. Therefore, we speculate that increases in viral concentration after long shoot tip culture may have an explanation contrary to the above mentioned reasons. Moreover, plant viruses only move among adjacent cells that share plasmodesmata connections or over long distances via the phloem. These spatial restrictions are potential bottlenecks for plant viruses in an infected plant, while long shoot tip culture breaks these restrictions [40]. This study used a novel strategy to study grapevine viruses, which should support future studies on plant viruses, especially those infecting woody plants, e.g., in vitro grafting experiments during evaluation of grapevine resistance to viruses, or environmentally secured long term maintenance of grapevine virus collections in vitro.

## 4. Materials and Methods

### 4.1. Plant Materials

New shoot tips (length about 3.0 cm) of six grapevine cultivars (Cabernet Franc, Cabernet Gernischt, Cabernet Sauvignon, Wink, Victoria, and Merlot) were collected from six regions of China in August 2021 (Table 1).

### 4.2. Establishment of In Vitro Cultures

**Figure 4:**
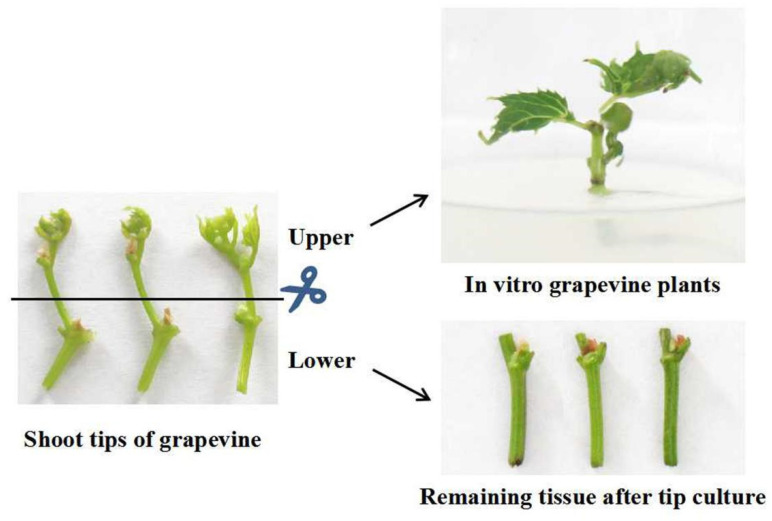
Sampling of long shoot tip culture.

Firstly, the shoot tips were cleaned under tap water, placed in sterilized glasses, and then sterilized by treating them with 75% ethanol and 0.1% mercury bichloride for 30 s and 15 min, respectively. After washing them with sterilized distilled water, the shoot tips were pruned to 1.5 cm in size and were inserted in modified 1/2MS solid medium [41]. These explants were incubated under a controlled environment (24 °C, 16 h photoperiod, and 2000 lx light intensity). The survival rates of the explants were investigated after 60 days, when the whole plants were regenerated. The presence and diversity of viruses in six grapevine cultivars was assessed before and after shoot tip culture; the virus titers in the lower and upper part of shoot tips and the residual tissue after collecting explants (the same tissue as the lower part of shoot tips) and in vitro grapevine plants were also analyzed (Figure 4). The in vitro grown plants were transferred to fresh MS medium at 45-day intervals.

### 4.3. RNA Extraction, cDNA Synthesis and Regular PCR Amplification

Total RNA was extracted from different materials of grapevine plants following the protocol described by Hu et al. [41]. First-strand cDNA was synthesized with M-MLV reverse transcriptase (Promega, Madison, WI, USA). A 25 μL PCR reaction was set up following the protocol described by Hu et al. [38] with specific virus primer pairs (Table 3). All these primer pairs have previously proven to be effective and polyvalent against grapevine virus variants present in China [25].

### 4.4. Cloning and Sequencing

The PCR products of grapevine viruses were purified using a PCR purification kit (Axygen, Hangzhou, China). Then, the purified products were inserted into the pTOPO-TA vector (Aidlab, Beijing, China) and transformed into Escherichia coli DH5α. For each grape cultivar, 13-17 positive clones selected from in vitro and in vivo materials were sequenced in both orientations. Multiple sequence alignment was conducted using the DNAMAN 5.2.2 program (Lynnon BioSoft, Vaudreuil, QC, Canada). To determine the variations during shoot tip culture, the mutation frequency/nucleotide (nt) of viral sequences was computed using the RT-PCR mutation frequency/nt subtracted from the uncorrected difference to yield total corrected mutation frequency/nt [35].

### 4.5. Quantitative Real-Time PCR (qPCR)

The RT Reagent Kit with gDNA Eraser (TaKaRa, Dalian, China) and SYBR^®^ Premix Ex Taq™ Tli RNaseH Plus (TaKaRa) were used in qPCR. The ΔCt method was used to calculate the relative virus titers in different plant samples. Each sample was analyzed in triplicate.

## Figures and Tables

**Table 1:** The survival and virus infection status of regenerated grapevine plants.

Cultivars	Origins	In Vivo		In Vitro	
		Infected Virus	No. of Dissected Tips	No. of Survival Tips (%)	Infected Virus
Cabernet Franc	Ningxia, China	GRSPaV, GRLaV-2	20	10 (50.0)	GRSPaV, GRLaV-2
Cabernet Gernischt	Liaoning, China	GRSPaV, GRLaV-3, GFkV	20	0	/
Cabernet Sauvignon	Tianjin, China	GRSPaV, GRLaV-2, GFkV	20	0	/
Wink	Guangxi, China	GRSPaV, GRLaV-2, GRLaV-3, GFkV	20	12 (60.0)	GRSPaV, GRLaV-2, GRLaV-3, GFkV
Victoria	Guangxi, China	GRSPaV, GRLaV-2, GRLaV-3, GFkV	20	14 (70.0)	GRSPaV, GRLaV-2, GRLaV-3, GFkV
Merlot	Beijing, China	GRSPaV	20	16 (80.0)	GRSPaV
Merlot	Sichuan, China	GRLaV-1, GVA	20	12 (60.0)	GRLaV-1, GVA

GRSPaV: grapevine rupestris stem pitting-associated virus; GLRaV-1: grapevine leafroll-associated virus 1; GLRaV-2: grapevine leafroll-associated virus 2; GLRaV-3: grapevine leafroll-associated virus 3; GFkV: grapevine fleck virus; GVA: grapevine virus A.

**Table 2:** Comparison of nucleotide identities and differences of grapevine viruses before and after shoot tip culture.

Infected Virus	Cultivars	No. of Clones Sequenced	Identities (%)			Consistency (%)			Mutation Frequency/nt			
			In Vivo	In Vitro	Between Group	In Vivo	In Vitro	Between Group	In Vivo	In Vitro	Uncorrected Difference	Corrected Difference ^a^
GRLaV-2	Wink	15	99.7–100.0	99.4–100.0	99.4–100.0	99.94	99.96	99.97	1.7 × 10^−4^	3.5 × 10^−4^	1.8 × 10^−4^	0
	Victoria	15	99.5–100.0	99.2–100.0	99.2–100.0	99.89	99.91	99.90	8.7 × 10^−4^	8.7 × 10^−4^	0	0
	Cabernet Franc	13	98.9–100.0	99.5–100.0	98.9–100.0	99.79	99.85	99.73	8.1 × 10^−4^	8.1 × 10^−4^	0	0
GRLaV-3	Wink	15	99.7–100.0	99.5–100.0	99.5–100.0	99.93	99.93	99.93	7.1 × 10^−4^	7.1 × 10^−4^	0	0
	Victoria	17	99.5–100.0	99.2–100.0	99.2–100.0	99.87	99.87	99.87	8.1 × 10^−4^	6.3 × 10^−4^	0	0
GRSPaV	Wink	16	99.2–100.0	99.2–100.0	99.1–100.0	99.89	99.88	99.83	6.2 × 10^−4^	7.0 × 10^−4^	0.8 × 10^−4^	0
	Merlot-B	16	99.8–100.0	99.6–100.0	99.7–100.0	99.94	99.93	99.93	9.7 × 10^−4^	9.7 × 10^−4^	0	0

^a^ Sporadic changes attributed to combined error (5.4 × 10^−4^/nt) were subtracted to yield the corrected mutation frequency/nucleotide.

**Table 3:** Primers used for grapevine virus detection.

Viruses	Primers	Sequences (5′-3′)	Size (bp)	Reference
Regular PCR				
GFkV	C1/R	TGGTCCTCGGCCCAGTGAAAGTA	344	[42]
	V1/R	GGCCAGGTTGTAGTCGGTGTTGTC		
GVA	H587	GACAAATGGCACACTACG	429	[43]
	C995	AAGCCTGACCTAGTCATCTTGG		
GRSPaV	RSP52	TGAAGGCTTTAGGGGTTAG	905	[44]
	RSP53	CTTAACCCAGCCTTGAAAT		
GRLaV-1	L1A	TCTTTACCAACCCCGAGATGAA	232	[45]
	L1B	GTGTCTGGTGACGTGCTAAACG		
GRLaV-2	L2HSPL	CARAAYAATTCGGCGTACAT	386	[46]
	L2HSPR	TAATTGGCRGGYACYGAACA		
GRLaV-3	LR3PU	CGCTCATGGTGAAAGCAGACG	653	[47]
	LR3PD	CTTAGAACAAAAATATGGAGCAG		
Quantitative real-time PCR				
GFkV	F1	TCAAGGACTCCGTCACCTACA	110	This study
	R1	AGGATGGAGCCGCAGAT		
GRSPaV	Y-cpf1	GCACGTCACTGCTCTGATGTTGG	170	[41]
	Y-cpr1	GTCTCCAGATGGATGTTCCACACGAT		
GRLaV-1	GLRaV-1F	GTGGAGAGTATGATTCCGTGGTCAC	267	[48]
	GLRaV-1R	CACTGGCACGTTAACTTGAGGTCG		
GRLaV-2	RL2 P19	CTAACAATTTCTTCTTTGGATCGCAT	155	[49]
	RL2 P24	AGAATGTCTTCAGCTTCATAAGGAG		
GRLaV-3	LR3-F1	GGGRACGGARAAGTGTTACC	143	[50]
	LR3-R1	TCCAAYTGGGTCATRCACAA		
Internal control	Vivi-18Sf	AAGCCCGATCCAGCAATA	176	[51]
	Vivi-18Sr	GCCCTTTACGCCCAGTCA		

## Data Availability

All data were presented in the manuscript, so the study did not report other data.
